# Current Experience in Testing Mitochondrial Nutrients in Disorders Featuring Oxidative Stress and Mitochondrial Dysfunction: Rational Design of Chemoprevention Trials

**DOI:** 10.3390/ijms151120169

**Published:** 2014-11-05

**Authors:** Giovanni Pagano, Annarita Aiello Talamanca, Giuseppe Castello, Mario D. Cordero, Marco d’Ischia, Maria Nicola Gadaleta, Federico V. Pallardó, Sandra Petrović, Luca Tiano, Adriana Zatterale

**Affiliations:** 1 Istituto Nazionale Tumori Fondazione G. Pascale—Cancer Research Center at Mercogliano (CROM)—IRCCS, Naples I-80131, Italy; E-Mails: a.aiello@istitutotumori.na.it (A.A.T.); beppe.castello@gmail.com (G.C.); 2 Research Laboratory, Dental School, Universidad de Sevilla, Sevilla 41009, Spain; E-Mail: mdcormor@us.es; 3 Department of Chemical Sciences, University of Naples “Federico II”, Naples I-80126, Italy; E-Mail: dischia@unina.it; 4 National Research Council, Institute of Biomembranes and Bioenergetics, Bari I-70126, Italy; E-Mail: marianicola.gadaleta@uniba.it; 5 CIBERER (Centro de Investigación Biomédica en Red de Enfermedades Raras), University of Valencia—INCLIVA, Valencia 46010, Spain; E-Mail: federico.v.pallardo@uv.es; 6 “Vinca” Institute of Nuclear Sciences, University of Belgrade, Belgrade 11001, Serbia; E-Mail: dragita.sandra@gmail.com; 7 Biochemistry Unit, Department of Clinical and Dental Sciences, Polytechnical University of Marche, Ancona I-60131, Italy; E-Mail: l.tiano@univpm.it; 8 Genetics Unit, Azienda Sanitaria Locale (ASL) Napoli 1 Centro, Naples I-80136, Italy; E-Mail: azatt@tin.it

**Keywords:** mitochondrial nutrients, mitochondrial dysfunction, oxidative stress, oxidative phosphorylation, Krebs cycle

## Abstract

An extensive number of pathologies are associated with mitochondrial dysfunction (MDF) and oxidative stress (OS). Thus, mitochondrial cofactors termed “mitochondrial nutrients” (MN), such as α-lipoic acid (ALA), Coenzyme Q10 (CoQ10), and l-carnitine (CARN) (or its derivatives) have been tested in a number of clinical trials, and this review is focused on the use of MN-based clinical trials. The papers reporting on MN-based clinical trials were retrieved in MedLine up to July 2014, and evaluated for the following endpoints: (a) treated diseases; (b) dosages, number of enrolled patients and duration of treatment; (c) trial success for each MN or MN combinations as reported by authors. The reports satisfying the above endpoints included total numbers of trials and frequencies of randomized, controlled studies, *i.e.*, 81 trials testing ALA, 107 reports testing CoQ10, and 74 reports testing CARN, while only 7 reports were retrieved testing double MN associations, while no report was found testing a triple MN combination. A total of 28 reports tested MN associations with “classical” antioxidants, such as antioxidant nutrients or drugs. Combinations of MN showed better outcomes than individual MN, suggesting forthcoming clinical studies. The criteria in study design and monitoring MN-based clinical trials are discussed.

## 1. Introduction

The functions of mitochondria, unconfined to bioenergetic pathways, were linked to OS by studies in early 1990’s reporting on reactive oxygen species (ROS) formation as by-products of oxygen metabolism [1,2]. The implications for OS in Parkinson’s disease were discovered by Di Monte *et al.* [3] and associated with MDF by a correlation of complex I–III deficiency with lower-than-normal levels of CoQ10, a cofactor in the oxidative phosphorylation (OXPHOS) pathway [4,5].

The association of OS and MDF has been reported in in mitochondrial diseases [6,7], and our recent reviews evaluated the literature showing that OS/MDF is involved in broad-ranging pathologies, including some genetic diseases [8], aging and age-associated disorders, neurologic and psychiatric diseases, malignancies and autoimmune diseases [9]. This state-of-art has prompted a growing body of literature from clinical studies, aimed at compensating OS/MDF by means of MN administration [10,11,12,13,14]. These endogenous cofactors, such as ALA, CoQ10 and CARN (or CARN derivatives) are essential in mitochondrial functions.

ALA exists in two redox states, and the reduced thiol form is a potent mitochondrial antioxidant, a metal chelator and a glutathione (GSH) repletor. It is involved as an essential cofactor in Krebs cycle for mitochondrial α-ketoacid dehydrogenases. CoQ10 is a benzoquinone derivative playing a central role in the mitochondrial respiratory chain through shuttling between three redox forms, the quinone, the semiquinone and the hydroquinone. It acts as a carrier accepting electrons from mitochondrial complex I and complex II and transferring them to complex III. CARN (bioactive l-form) is biosynthesized from lysine and methionine and is concentrated in tissues that use fatty acids as their primary source of energy. It is involved in the transport of long-chain fatty acids from the intermembraneous space to the matrix in the mitochondria for generation of metabolic energy.

A number of studies have demonstrated MN deficiencies in several diseases [9], either related to deficiencies in OXPHOS activities [15,16,17,18] or caused by their degradation due to OS-related by-products as, e.g., by ALA oxidative modification induced by 4-hydroxynonenal in Alzheimer disease brain [19]. The present review is to provide an overall survey of the currently published clinical trials having tested each of the above MN, or their combinations, and/or their associations with “typical” antioxidants, including antioxidant nutrients and/or herbal preparations and/or disease-specific drugs. For each disease, or disease group, the total numbers of clinical trials were recorded, along with the relative frequencies of controlled, randomized trials. No further attempt was made to evaluate the clinical and/or laboratory outcomes of the evaluated reports. The major objective of the present survey was providing an overall state-of-art, and suggesting some prospects in study design and in monitoring the outcomes of forthcoming clinical trials. These should be aimed at verifying any specific MN deficiency(ies) associated to a given disorder, and at prompting adequate follow-up in monitoring the effects, if any, of mitochondria-targeted interventions.

## 2. Methods

A MedLine retrieval up to July 2014 was carried out for each MN. The papers reporting on clinical trials for each MN were evaluated according to: (a) treated diseases (or disease groups); (b) dosages, number of enrolled patients and duration of treatment; (c) numbers of trials and frequencies of randomized, controlled studies (opposed to open-label and pilot studies); and (d) an empyrical “success ratio” (SR) of trials testing each of the individual MN or their combinations, as reported by authors; SR was calculated by dividing the number of successful results by the total of trials for each agent. No clinical trials involving healthy volunteers were included for evaluation. The reports failing to provide clear-cut data for dosages, the numbers of patients and/or treatment duration were not included in survey, nor were included self-repeating reports of previous or contemporary studies.

## 3. α-Lipoic Acid

As shown in Table 1, ALA testing was reported in 81 evaluated clinical trials on a total of 2980 patients, since the pioneering report by Marshall *et al.* on alcohol-related liver disease [10], and since early studies in 1990’s in counteracting diabetes-associated neuropathies [20,21,22]. Both Type 1 and Type 2 diabetes mellitus (DM) have been the major focus for ALA administration, encompassing 42 trials (of which 30 controlled trials) on a total of 2980 patients and with a SR = 0.93 [20,21,22,23,24,25,26,27,28,29,30,31,32,33,34,35,36,37,38,39,40,41,42,43,44,45,46,47,48,49,50,51,52,53,54,55,56,57,58,59,60,61]. A major outcome of ALA treatment in diabetic patients consisted of the amelioration of neurologic damage [22,23,24,26,27,28,29,30,31,33,34,35,37,39,42,46,49,51,52,60,61]. Moreover, ALA-treated diabetic patients displayed decreased serum concentrations of thiobarbituric acid reactive substances (TBARS) [20,43], increased insulin sensitivity [21,25,36,58], an improvement in endothelium function [38,43,55], decreased erectile dysfunction [59] and decreased urinary PGF2α-isoprostanes, a marker of oxidative damage [50].

Beyond the literature of ALA-centered clinical trials in DM, a broad clinical use of ALA is recognized as a prescription generic drug for DM in Germany (German Drug Index), thus the use of ALA in diabetic patients may be seen as an established practice in the specialist community.

**Table 1:** Clinical studies utilizing α-lipoic acid aimed at compensating oxidative stress (OS)/mitochondrial dysfunction (MDF)-related pathogenetic mechanisms.

Diseases/Conditions	No. Studies (Controlled Studies)	No. Treated Patients	Success Ratio	References
Type 1 and Type 2 diabetes	42 (30)	2980	0.93	[20,21,22,23,24,25,26,27,28,29,30,31,32,33,34,35,36,37,38,39,40,41,42,43,44,45,46,47,48,49,50,51,52,53,54,55,56,57,58,59,60,61]
Neurological diseases	9 (5)	509	0.89	[62,63,64,65,66,67,68,69,70]
Liver and metabolic diseases	8 (5)	417	0.86	[10,71,72,73,74,75,76,77]
Heart and vessel diseases	4 (4)	137	1.00	[78,79,80,81]
Kidney diseases	4 (3)	288	0.25	[82,83,84,85]
Genetic and mitochondrial diseases	3 (2)	129	0.33	[86,87,88]
Burning mouth syndrome	5 (5)	293	0.60	[89,90,91,92,93]
Other diseases ^§^	6 (4)	525	1.00	[94,95,96,97,98,99]
Total	81	5278		

^§^ Malignancies [94,95]; HIV [96,97]; vitiligo [98]; osteoporosis [99].

Other clinical trials successfully tested ALA in neurological diseases [62,63,64,65,66,67,68,69,70], in liver and metabolic diseases [10,71,72,73,74,75,76,77], and in heart and vessel diseases [78,79,80,81] (Table 1).

Lesser, if any, positive effects were reported for three ALA trials in kidney diseases [82,83,84,85]. Other clinical trials tested the effects of ALA administration in genetic and mitochondrial diseases [86,87,88], burning mouth syndrome [89,90,91,92,93], cancer cachexia [94,95], mitochondrial function in HIV-1-related lipoatrophy [96,97], in vitiligo [98], and in osteoporosis [99].

Among these studies, some reports investigated the comparative effects of different MN formulations, and/or provided evidence for combined effects in clinical and laboratry endpoints.

Li *et al.* [79] showed that ALA administration to patients with acute coronary syndrome caused a decrease in an OS marker, 8-*iso*-prostaglandin F2α and an increase in aldehyde dehydrogenase-2, responsible for acetaldehyde oxidation in ethanol metabolism that also provides protection against OS. Protective effects were reported by Martins *et al.* [87] in sickle cell trait subjects and sickle cell patients following ALA administration, with a significant increase in blood catalase (CAT) and a significant decrease in malondialdehyde (MDA) and in carbonyl levels.

A clinical trial reported by Galasko *et al.* [64] tested the effects of combined ALA with Vit E and Vit C (E/C/ALA), or CoQ10, or placebo in three groups of patients with Alzheimer's disease, finding a decrease in CSF F2-isoprostane levels in the E/C/ALA group that suggested a reduction of OS in the brain. However, this treatment raised the caution of faster cognitive decline.

Altogether, the current body of evidence may suggest further interventions with ALA in several disorders, beyond DM and the other disorders where ALA treatment led both to clinical improvements and to compensation of OS-related endpoints.

## 4. Coenzyme Q10

A recent review has focused on the multiple implications of CoQ10 deficiency and of CoQ10 administration encompassing an extensive number of disorders [100]. Out of 101 reports on clinical trials testing CoQ10, 39 studies were focused on support to patients with heart and vessel disorders or undergoing heart surgery [11,12,81,101,102,103,104,105,106,107,108,109,110,111,112,113,114,115,116,117,118,119,120,121,122,123,124,125,126,127,128,129,130,131,132,133,134,135,136], including 32 controlled studies, as shown in Table 2. The pioneering studies by the groups of Tanaka [11] and of Langsjoen [12] since 1982 provided the avenue for a number of clinical trials assessing the successful outcomes of CoQ10 administration to patients with coronary artery disease, cardiomyopathy, heart failure, or heart surgery, with an overall SR = 0.89. Among these studies, Dai *et al.* [105] evaluated mitochondrial function in patients with ischemic left ventricular systolic dysfunction receiving CoQ10 *vs.* placebo, in terms of plasma lactate/pyruvate (LP) ratio. After an 8-week treatment, CoQ10-treated patients had significant increases in plasma CoQ10 concentration, brachial flow-mediated dilation, and decreased LP ratio, showing positive effects both on heart function and in balancing mitochondrial activities [105]. Lee *et al.* [106] tested CoQ10 administration in patients with coronary artery disease and found a decrease in the inflammatory markers C-reactive protein (hs-CRP) and interleukin 6 (IL-6), and decreased MDA and superoxide dismutase (SOD) activities, along with increased CoQ10 levels. Direct evidence for a CoQ10-induced compensation of mitochondrial function was reported by Rosenfeldt *et al.* [133] in patients undergoing heart surgery, which resulted in increased CoQ10 levels in serum, atrial trabeculae, and isolated mitochondria compared with patients receiving placebo, with an improvement in mitochondrial respiration, as adenosine diphosphate/oxygen ratio, and a decrease in mitochondrial MDA content.

**Table 2:** Clinical studies utilizing coenzyme Q10 aimed at compensating OS/MDF-related pathogenetic mechanisms.

Diseases/Conditions	No. Studies (Controlled Studies)	No. Treated Patients	Success Ratio	References
Heart and vessel diseases	39 (32)	3386	0.89	[11,12,81,101,102,103,104,105,106,107,108,109,110,111,112,113,114,115,116,117,118,119,120,121,122,123,124,125,126,127,128,129,130,131,132,133,134,135,136]
Genetic and mitochondrial diseases	18 (8)	680	0.75	[13,14,88,137,138,139,140,141,142,143,144,145,146,147,148,149,150,151]
Neurological diseases	16 (10)	1185	0.87	[65,152,153,154,155,156,157,158,159,160,161,162,163,164,165,166]
Type 1 and Type 2 Diabetes	9 (7)	370	0.89	[41,166,167,168,169,170,171,172,173,174]
Malignancies	6 (2)	301	0.67	[175,176,177,178,179,180]
Kidney diseases	4 (2)	171	0.50	[181,182,183,184]
Other diseases ^§^	15 (13)	555	0.59	[76,185,186,187,188,189,190,191,192,193,194,195,196,197,198]
Total	107	6648		

^§^ Psoriasis [76,196]; metabolic syndrome [185,186,187]; statin-induced myalgias [188,189,190,191,192]; bronchial asthma [193]; pre-eclampsia [194]; mucocutaneous infections [195]; cataract surgery [197]; idiopathic infertility [198].

Eighteen studies (of which 8 controlled studies) have focused on clinical trials testing CoQ10 in a number of genetic and mitochondrial diseases [13,14,88,137,138,139,140,141,142,143,144,145,146,147,148,149,150,151] on 680 patients (Table 2) with different success across the different diseases investigated (SR = 0.75).

Among these studies, Friedreich ataxia (FRDA) is relevant for a deficiency in the gene encoding frataxin, a mitochondrial protein implicated in iron metabolism and glutathione balance [139], and lower-than-normal CoQ10 levels [139,140]. FRDA was investigated by Cooper’s group [140,141] for CoQ10 and Vit E administration finding a significant improvement in cardiac and skeletal muscle bioenergetics and in International Co-operative Ataxia Ratings Scale. Rodriguez *et al.* [88], as mentioned above, tested the effect of a combination therapy with CoQ10 with ALA and creatine monohydrate on several outcome variables in patients with mitochondrial diseases, both achieving amelioration of clinical and of OS/MDF endpoints [88]. Limited evidence for a CoQ10-induced improvement in brain and muscle bioenergetics in patients with mitochondrial diseases was reported by Barbiroli *et al.* [142] and by Glover *et al.* [145].

A set of studies was focused on 16 clinical trials (of which 10 controlled trials) testing CoQ10 supplementation in 1185 patients with eight neurologic disorders [65,152,153,154,155,156,157,158,159,160,161,162,163,164,165,166].

The above-mentioned report by Galasko *et al.* [65] tested CoQ10 as an alternative treatment to E/C/ALA in patients with Alzheimer’s disease, failing to report any effects of CoQ10, unlike E/C/ALA. A study by Shults *et al.* [152] in patients with Parkinson’s disease (PD) tested the effects of three CoQ10 dosages (300, 600, or 1200 mg/d) that were evaluated with the Unified Parkinson Disease Rating Scale at the baseline and up to 16-month visits. Less disability developed in subjects assigned to CoQ10 than in those assigned to placebo, with a significant benefit in patients receiving the highest dosage [152]. Moderate beneficial effects of CoQ10 in PD patients were also reported by Müller *et al.* [153], though at the low dosage (360 mg/d) reported by Shults *et al.* [152]. A study by Storch *et al.* [154] found that nanoparticular CoQ10 at a dosage of 300 mg/d was well tolerated, though failing to display symptomatic effects in midstage PD.

Sanoobar *et al.* [155,156] tested CoQ10 in multiple sclerosis (MS) patients, and found reduced OS and increased antioxidant enzyme activity [155], with a significant decrease of tumor necrosis factor alpha (TNF-α) and IL-6 levels [156] in the CoQ10 group compared to placebo group.

A series of studies focused on the role of CoQ10 in fibromyalgia (FM) in long-term treatment of fibromyalgic patients, resulting in significant pain reduction, fatigue, and morning tiredness [157,158,159,160]. CoQ10-treated patients underwent significant reduction in the pain visual scale and in tender points, recovery of inflammation, antioxidant enzymes, mitochondrial biogenesis, and AMPK gene expression levels [157,158]. A recent report by Miyamae *et al.* [159] measured plasma levels of ubiquinone-10, ubiquinol-10, free cholesterol, cholesterol esters, and free fatty acids in patients with juvenile FM *vs.* healthy control subjects. Plasma level of ubiquinol-10 was significantly decreased and the ratio of ubiquinone-10 to total CoQ10 was significantly increased in juvenile FM relative to healthy controls, with compensated plasma levels of lipid endpoints [159]. A previous uncontrolled trial by Lister [160] tested the effects of combined CoQ10 and and *Ginkgo biloba* extract reporting improved quality-of-life in FM patients. Altogether, independent studies support the usefulness of CoQ10 in FM pathogenesis and point to CoQ10 utilization in FM both in inducing clinical improvements and in compensating FM-associated OS/MDF.

No evidence of CoQ10-induced beneficial effects were found in a Phase II clinical trial in patients with amyotrophic lateral sclerosis [161].

Migraine has been investigated for deficiency of CoQ10, which showed lower-than-normal levels in patients with pediatric and adolescent migraine [162]. Clinical trials testing CoQ10 in migraine patients provided overall positive, though apparently transient outcomes [162,163,164].

Forester *et al.* [165] tested high-dose CoQ10 in patients with geriatric bipolar depression suggesting a reduction in depression symptom severity.

Stamelou *et al.* [166] tested CoQ10 in patients with progressive supranuclear palsy (PSP), and found that patients receiving CoQ10 displayed decreased concentration of low-energy phosphates, with increased ratio of high-energy phosphates to low-energy phosphates. Clinically, the PSP rating scale and the Frontal Assessment Battery improved upon CoQ10 treatment compared to placebo [166].

Nine clinical trials (seven controlled trials) were conducted by testing CoQ10 in 370 patients with Type 1 and Type 2 DM, particularly focusing on DM-associated vascular complications and on statin-induced CoQ10 depletion [167,168,169,170,171,172,173,174]. The outcomes of these studies showed beneficial effects of CoQ10, better if combined with fenofibrate [169]. By considering the established advantages of ALA treatment in DM patients [20,21,22,23,24,25,26,27,28,29,30,31,32,33,34,35,36,37,38,39,40,41,42,43,44,45,46,47,48,49,50,51,52,53,54,55,56,57,58,59,60,61], one may suggest further clinical trials toward the clinical use of ALA and CoQ10 combinations in DM. This trial design was performed by Palacka *et al.* with successful outcomes both in improving heart left ventricular function and in decreasing lactate dehydrogenase activity in DM patients treated with ALA and CoQ10 [41].

Six clinical trials have been reported testing CoQ10 in association with other agents (e.g., riboflavin and niacin) in patients with advanced malignancies under antineoplastic therapy as supportive or palliative treatments [175,176,177,178,179,180]. Some of these trials reported survival prolongation [177], and an early study by Lockwood *et al.* [176] reported partial and complete regression of breast cancer in patients treated with CoQ10. A clinical trial by Rusciani *et al.* [180] tested the effects of CoQ10 and interferon α-2b in melanoma patients, and found significantly decreased recurrence rates *vs.* patients receiving interferon only.

Four clinical trials tested the CoQ10 in patients with chronic kidney disease or undergoing hemodialysis, or submitted to statin treatment [181,182,183,184]. Sakata *et al.* [182] reported CoQ10 administration in hemodialysis patients as partially effective for suppressing OS. Shojaei *et al.* [184] tested CoQ10 and CARN, separately or in association, in hemodialysis patients who were on statin treatment. This study showed that supplementation with CoQ10 and CARN reduced serum levels of lipoprotein(a) in maintenance hemodialysis patients treated with statins. Altogether, the clinical trials testing CoQ10 in patients with kidney diseases failed to report clear-cut beneficial effects (SR = 0.50).

Controversial results were reported from clinical trials aimed at testing CoQ10 in counteracting various dysmetabolic conditions [185,186,187], statin-induced myalgias [188,189,190,191,192], bronchial asthma [193], pre-eclampsia [194], cutaneous infections [195], psoriasis [76,196], cataract surgery [197], and idiopathic infertility [198].

## 5. l-Carnitine and Acetyl- or Propionyl-Carnitine

Multi-decade long investigations showed the crucial roles of CARN in mitochondrial physiology, along with CARN deficiency in several disorders. As reviewed by Gilbert since 1985 [199], CARN deficiency results in accumulation of neutral lipid deposits within skeletal muscle, myocardium and liver, with mitochondria aggregates in skeletal muscle and myocardium. The efficacy of CARN *vs.* acetyl-CARN (ALC) was compared both *in vitro* and in aging rat brain since early studies [200,201]. CARN and ALC were similar in elevating carnitine levels in plasma and brain as well as in increasing ambulatory activity of old rats. However, ALC but not CARN was able to decrease the level of oxidative stress biomarkers in the brain of old rats [201]. Subsequent investigations pointed to a role of ALC in the reactivation of mitochondrial biogenesis in aging through the increased expression of PGC-1α signaling pathway [202,203].

As shown in Table 3, out of 74 evaluated studies, 18 clinical trials (of which 16 controlled trials) tested the effects of CARN in kidney diseases, treating a total of 427 patients with end-stage renal disease under hemodialysis, based on the rationale that CARN levels sharply decrease during hemodialysis, thus impairing response to recombinant human erythropoietin (rHuEPO) [181,184,204,205,206,207,208,209,210,211,212,213,214,215,216,217,218,219,220]. One study utilized both CARN and CoQ10—separately or in association—finding positive effects of both supplements, yet without evidence for improvement effects following combined supplementation [184]. Some reports failed to find significant improvements following CARN administration [205,206,207,208,213,220] (overall SR = 0.58). One might note that out of four trials adopting *i.v*. CARN (or PLC) administration, three resulted in significant improvements in the tested endpoints, such as Medical Outcomes Short Form-36, rHuEPO requirement, hemodynamic flow, endothelial profile and homocysteine levels, or inflammatory status as significant decrease in C-reactive protein [210,211,217].

**Table 3:** Clinical studies utilizing l-carnitine (CARN) (or acetyl-CARN (ALC)) aimed at compensating OS/MDF-related pathogenetic mechanisms.

Diseases/Conditions	No. Studies (Controlled Studies)	No. Treated Patients	Success Ratio	References
Kidney diseases	18 (16)	427	0.58	[184,204,205,206,207,208,209,210,211,212,213,214,215,216,217,218,219,220]
Type 1 and Type 2 Diabetes	13 (9)	1894	1.00	[221,222,223,224,225,226,227,228,229,230,231,232,233]
Heart and vessel diseases	9 (6)	359	1.00	[78,114,234,235,236,237,238,239,240]
Liver diseases	6 (2)	275	1.00	[241,242,243,244,245,246]
Neurological diseases	9 (9)	384	1.00	[68,247,248,249,250,251,252,253,254]
Malignancies	7 (6)	699	0.70	[255,256,257,258,259,260,261]
Genetic diseases	6 (4)	202	1.00	[262,263,264,265,266,267]
HIV	4 (1)	93	1.00	[97,268,269,270]
Other diseases ^§^	2 (2)	99	1.00	[271,272]
Total	74	4432		

^§^ Major surgery [271]; ulcerative colitis [272].

Unlike renal diseases, all of 13 clinical trials (9 controlled trials) supplementing CARN (or ALC, or PLC) to patients with Type 1 and Type 2 DM to a total of 1894 patients succeeded achieving improvements in disease status [221,222,223,224,225,226,227,228,229,230,231,232,233]. Long-term infusion of CARN or ALC in DM patients was tested by the group of Giancaterini [221,222], resulting in improved insulin sensitivity, and in decreased lactate levels, suggesting activation of pyruvate dehydrogenase, whose activity is depressed in the insulin resistant status. Three independent studies found a long-term treatment with ALC or with PLC effective and well tolerated in improving neurophysiological parameters and in reducing pain [223,226,229]. Derosa *et al.* reported that CARN significantly lowered the plasma lipoprotein(a) *vs.* placebo in hypercholesterolemic patients with DM [224]. A clinical trial with PLC in patients with DM and peripheral arterial disease was reported by Ragozzino *et al.* [225], who found significant increases in maximal walking distance, initial claudication distance, and a decrease in dosage of oral antihyperglycemic agents. Two studies investigated the effects of CARN or ALC treatment on lipidemic profile of DM patients [223,224], failing to detect a decrease of lipidemic profile of CARN alone to this endpoint, yet Solfrizzi *et al.* [228] found significant effects from combined treatment with CARN and simvastatin in lowering Lp(a) serum levels in patients with DM than with simvastatin alone. A study by Malaguarnera *et al.* [230] testing CARN in DM patients found significant improvements compared with the placebo group in a number of OS endpoints, proinflammatory markers and lipidemic profile.

A clinical trial was conducted by Ruggenenti *et al.* [231] by testing the effects of ALC on glucose disposal rate in patients with metabolic syndrome and hypertension, reporting that ALC ameliorated arterial hypertension, insulin resistance, glucose tolerance, and hypoadiponectinemia. Patients with Type 1 DM were treated by Uzun *et al.* [233] with CARN to evaluate changes in their neuropathy frequency and nerve conduction velocity, and found that CARN treatment in early stages improved neuropathy in Type 1 DM patients.

Altogether, the body of literature of clinical trials testing CARN—or CARN derivatives—in patients with DM provides substantial evidence for the amelioration of multiple DM-related endpoints that encourage the clinical use of CARN—or CARN derivatives—in DM patients. One may note that no report was found of combined treatments of DM patients using CARN with either ALA or CoQ10, despite the body of positive evidence accumulated in clinical trials testing ALA, or CoQ10 in DM patients (as shown Table 1 and Table 2).

A series of 9 clinical trials (6 controlled trials) were reported testing CARN (or ALC, or PLC) in 359 patients with heart and vessel diseases [78,114,234,235,236,237,238,239,240]. A study by McMackin *et al.* [78] reported on the effects of combined ALC and ALA treatment in patients with coronary artery disease, and found significant improvement as increased brachial artery diameter, a trend to decreased systolic blood pressure for the whole patient group, with a significant effect in the subgroup with blood pressure above the median and in the subgroup with metabolic syndrome. An analogous investigation was reported by Kumar *et al.* [114], who tested the effects of carni Q-gel (CARN and ubiquinol) in patients with heart failure. Serum concentration of IL-6 was significantly decreased in the intervention group without such changes in the control group. TNF-α, which was comparable at baseline, also showed a greater decline in the carni Q-gel group compared to the placebo group. Serum CoQ10 showed a significant increase in the carni Q-gel group as compared to the control group. The symptom scale indicated that the majority of treated patients showed a significant improvement compared to the placebo group [114]. Altogether, both of these clinical trials using associations of ALC with ALA [78] or CARN with CoQ10 (as ubiquinol) [114] showed definite improvements both in heart and vessel performance and in related laboratory endpoints.

Relevant mechanistic data on PLC treatment in patients with peripheral arterial disease (PAD) were obtained by Loffredo *et al.* [234], who measured serum levels of nitrite and nitrate (NO_x_) and 8-hydroxy-2'-deoxyguanosine (8-OHdG), and maximal walking distance (MWD) in PAD patients. Serum levels of 8-OHdG were significantly increased in PAD patients, and serum levels of NO_x_ were significantly decreased. Patients treated with PLC showed a significant increase of MWD and in NO_x_, while 8-OHdG levels underwent a significant decrease, unlike the patients given placebo [234].

Improved short-term exercise capacity, diastolic function and symptoms, WHO heart functional class were reported in patients receiving CARN [237,238], with improvements in diastolic function and in myocardial hypoxanthine concentration [239,240].

A total of 275 patients affected by various liver disorders were treated with CARN or ALC in 6 clinical trials (of which 2 controlled studies) [241,242,243,244,245,246]. Łapiński and Grzeszczuk [241] tested CARN (or l-ornitine l-aspartate) in patients with liver cirrhosis, and evaluated the outcomes as changes in serum concentrations of ammonia, cholesterol and triglycerides. A significant improvement was observed both in the patients treated with CARN and in those treated with l-ornitine l-aspartate. However, the individuals treated with CARN showed a significant increase of serum cholesterol and triglyceride concentrations.

Patients with non-alcoholic fatty liver disease were treated with CARN by Lim *et al.* [242]; by measuring a liver function test, peripheral blood mitochondrial DNA and 8-oxo-dG analysis. The results showed a decrease in ALT, AST, and total bilirubin in treated patients, not in controls. In the CARN treated patients, mitochondrial DNA copy number was significantly increased, unlike the control group. 8-oxo-dG levels showed a non-significant decrease in CARN group while it tended to increase in the control group. The group of Malaguarnera *et al.* has conducted a series of clinical trials testing the effects of CARN in patients with hepatic encephalopathy or with HCV-induced chronic hepatitis [243,244,245,246]. Beneficial effects in CARN-treated patients included significant differences in AST, ALT, viremia, Hb, RBC, WBC and platelets. Also significant improvements were detected in neurologic scores and fatigue symptoms in hepatic encephalopathy following CARN administration [244,245].

Nine controlled clinical trials for CARN—mostly ALC—were conducted in a total of 384 patients with some neurological disorders, including Alzheimer's disease, multiple sclerosis, migraine, sciatica, fibromyalgia, chronic fatigue syndrome, and amyotrophic lateral sclerosis [68,247,248,249,250,251,252,253,254]. A report by Memeo and Loiero [68] compared the effects of treating sciatica patients with either ALA or ALC, and found significant protective effects from both of these agents detected as improvements from baseline in neuropathy on electromyography.

Tomassini *et al.* [247] tested the efficacy of ALC *vs.* amantadine, a widely used drug in treating patients with multiple sclerosis (MS)-related fatigue. The results showed significant effects of ALC compared with amantadine for the Fatigue Severity Scale, suggesting that ALC is better tolerated and more effective than amantadine for the treatment of MS-related fatigue.

Patients with Alzheimer’s disease and vascular dementia were treated with ALC by Gavrilova *et al.* [248], and the outcomes were assessed with MMSE and CGI scales, and a battery of neuropsychological tests. The treatment effect of ALC was significantly higher than in the placebo group.

Tarighat Esfanjani *et al.* [249] evaluated the effects of magnesium, CARN, and concurrent magnesium-CARN supplementation in migraine prophylaxis. Migrainous patients were randomly assigned into three intervention groups: magnesium oxide *vs.* CARN *vs.* Mg-CARN, and a control group. The results showed a significant reduction in all migraine indicators in all studied groups, yet without a clear-cut difference in-between the three supplementation regimens.

Four independent clinical trials tested CARN effects [250,251,252,253] in patients with chronic fatigue syndrome (CFS), or fibromyalgia (FM), or narcolepsy (NL). Pistone *et al.* [250] treated elderly subjects with onset of fatigue following slight physical activity. Wessely and Powell fatigue scores decreased significantly in subjects taking CARN *vs.* the placebo group, with significant improvements in total fat mass, total muscle mass, total cholesterol, LDL-C, HDL-C, triglycerides, apoA1, and apoB [250]. Miyagawa *et al.* [251] tested CARN administration in NL patients and found a significant reduction of excessive daytime sleepiness in CARN-treated NL patients *vs.* those receiving placebo. A clinical trial testing the effects of ALC in FM patients was reported by Rossini *et al.* [252]. The “total myalgic score” and the number of positive tender points declined significantly in the ALC-treated patients *vs.* the placebo group, and a significant difference was observed for depression and musculo-skeletal pain [252]. The effects of ALC and PLC were tested in patients with CFS by Vermeulen and Scholte [253]. This clinical trial compared ALC, PLC, and their combination. Clinical global impression of change after treatment showed considerable improvement in 59% of the patients in the ALC group and 63% in the PLC group, but less in the ALC plus PLC group (37%). In the ALC group the changes in plasma CARN levels correlated with clinical improvement [253].

A clinical trial by Beghi *et al.* tested the effects of ALC plus riluzole *vs.* riluzole and placebo on disability and mortality of patients with amyotrophic lateral clerosis [254]. Patients receiving ALC became non-self-sufficient to significantly less extent compared to those receiving placebo, and median survival was significantly extended in ALC-treated patients *vs.* placebo group [254].

Based on CARN depletion in advanced cancer, palliative treatments by CARN supplementation have been proposed by a series of seven clinical trials, of which six controlled trials [255,256,257,258,259,260,261]. Unfortunately, the outcomes showed a treatment-related increase of CARN serum levels, yet CARN or ALC supplementation did not improve fatigue in patients, or even increased chemotherapy-induced peripheral neuropathy [261]; otherwise, positive results were associated with treatments with multiple agents [258,259], thus casting doubts as to the specific efficacy of CARN supplementation.

A few genetic disorders were the focus of six clinical trials, of which 4 controlled trials, using CARN or ALC [262,263,264,265,266,267]. A clinical trial was carried out by Schöls *et al.* [262] by testing the administration of CARN *vs.* creatine in patients with Friedreich’s ataxia. After CARN treatment, patients had a significantly improved phosphocreatine recovery compared to baseline, while creatine effects did not reach significance.

Two independent clinical trials tested the effects of CARN in thalassemic patients, concurring to report improved cardiac function [263,264]. Moreover, Karimi *et al.* [264] tested the effect of combination therapy of hydroxyurea with CARN and magnesium chloride on hematologic parameters and cardiac function of patients with β-thalassemia intermedia. Patients were randomly divided into four groups: group A (hydroxyurea alone); group B (hydroxyurea and CARN); group C (hydroxyurea and magnesium chloride); and group D (hydroxyurea, CARN and magnesium chloride). In groups B, C, and D, mean Hb and hematocrit significantly increased during 6-month treatment [264].

Three independent studies tested the effects of CARN or ALC in patients with autistic spectrum disorders (ASD) [265,266,267]. Ellaway *et al.* [265] tested CARN in a group of Rett syndrome females, and found significantly improved sleep efficiency and expressive speech, compared to control Rett syndrome patients. A multi-center clinical trial testing ALC on the attention deficit hyperactivity disorder in fragile X syndrome boys was reported by Torrioli *et al.* [266]. Patients treated with ALC, compared with the placebo group, showed a stronger reduction of hyperactivity and improvement of social behavior [266]. Geier *et al.* [267] tested CARN in patients with ASD that were randomly assigned to receive a controlled regimen liquid CARN or placebo for 3 months. Treated patients showed significant improvements in Childhood Autism Rating Scale, modified clinical global impression, and Autism Treatment Evaluation Checklist scores.

Four clinical trials (of which one controlled trial) have tested the CARN, or ALC, aimed at improving different complications of HIV infection [97,268,269,270]. The above-cited report by Milazzo *et al.* [97] tested ALC *vs.* a combination of ALA and *N*-acetylcysteine (NAC) for a number of OS/MDF and other endpoints in HIV-1-infected patients with lipoatrophy, showing that either ALC, or ALA/NAC supplementation exerts a protective role on mitochondrial function in HIV-1-infected patients [97]. Two closely related studies [268,269] tested the effects of ALC in mitigating the neuropathy associated with antiretroviral toxicity in HIV-infected patients. Mean pain intensity score was significantly reduced, while electrophysiological parameters did not significantly change during treatment [269].

A controlled clinical trial was reported by Pignatelli *et al.* [271] on the effect of CARN on OS and platelet activation after major surgery, whose trauma is associated with an increased ROS production. At baseline and after treatment, OS was evaluated by detection of circulating levels of soluble NOX2-derived peptide, and by analyzing platelet ROS formation. The results showed an increase of OS endpoints in the placebo group compared with the baseline after the surgical intervention, while the CARN-treated group did not significantly differ from the baseline.

Patients with ulcerative colitis (UC) were treated by Mikhailova *et al.* [272] by PLC testing a clinical/endoscopic response. Patients with mild-to-moderate UC receiving stable oral aminosalicylate or thiopurine therapy were randomised to receive PLC or placebo. A significant response was found in patients receiving PLC as clinical/endoscopic response *vs.* those receiving placebo [272].

## 6. Treatments with Mitochondrial Nutrient Combinations

Out of 262 clinical trials reviewed here (Table 1, Table 2 and Table 3), only a tiny minority of seven reports referred to the combined use of two MN, ALA and/or CoQ10 and/or CARN [41,68,76,78,81,88,184], as shown in Table 4. Only four clinical trials tested the ALA/CoQ10 combination [41,76,81,88], while three clinical trials tested CARN combined with either ALA or CoQ10 [68,78,184]. Surprisingly, no retrieved report mentioned the combination of the three cofactors in any clinical trial; to the best of our knowledge; only Mattiazzi *et al.* [273] reported on the combined *in vitro* use of ALA, CoQ10 and CARN in improving a mitochondrial (mtDNA T8993G (NARP)) mutation on T8993G mutated cells.

**Table 4:** Clinical studies utilizing multiple mitochondrial nutrients (α-lipoic acid and/or coenzyme Q10 and/or l-carnitine) aimed at compensating OS/MDF-related pathogenetic mechanisms.

Diseases/Conditions	α-Lipoic Acid	Coenzyme Q10 (Daily Dose, mg/d)	l-Carnitine	References
Heart and vessel diseases	100	100	–	[81]
	400	–	1000	[78]
Mitochondrial diseases	600	240	–	[88]
Type 2 diabetes	100	60	–	[41]
Psoriasis	150	50	–	[76]
Sciatalgia	600	–	1180	[68]
Kidney diseases	–	100	500	[184]

The report by Rodriguez *et al.* [88] tested the combined use of ALA, CoQ10 and creatine in a group of patients with different mitochondrial diseases; these may be viewed as the “ideal” target for administration of MN, in view of supporting known MDF [6,7,9,14,274,275,276] that involve OS, decreased ATP production, and involvement of anaerobic energetic pathways. The combination therapy resulted in lower resting plasma lactate and OS markers (urinary 8-isoprostanes), and improved energetic performance [88].

Beyond mitochondrial diseases, a substantial body of evidence has been accumulated pointing to the involvement of OS/MDF in a number of heterogeneous diseases [9].

The combined use of ALA and CoQ10 was tested in patients with Type 2 DM [41] and in cardiac surgery patients [81]. One may note that both the report by Palacka *et al.* [41] and that by Leong *et al.* [81] fulfilled the expected clinical outcomes, along with the finding of decreased plasma lipid peroxides [41].

A number of clinical trials have been conducted by testing MN in combinations with classical antioxidants and/or herbal compounds and/or disease-specific drugs.

A total of 27 reports were evaluated concerning the therapies utilizing ALA or CoQ10 combinations with various antioxidants and/or herbal compounds and/or drugs (Table 5 and Table 6). As shown in Table 5, a study utilizing ALA and Vit E alone or in combination failed to find changes in the lipid profile or insulin sensitivity of Type 2 DM patients [44]. Supplementation of quercetin plus Vit C or ALA alone did not change the blood biomarkers of inflammation and disease severity of rheumatoid arthritis patients under conventional treatments [64].

A combined administration with ALA, CoQ10, magnesium orotate, ω-3 polyunsaturated fatty acids (ω-3 PUFA) and selenium was associated by Leong *et al.* [81] with improved redox status and reduced myocardial damage in patients undergoing heart surgery.

A clinical trial in Alzheimer’s disease patients showed that supplementation with ALA, Vit E and Vit C reduced OS in the brain [65]. The beneficial effects of combination therapy were also observed in Parkinson’s disease (PD) patients receiving homocysteine-lowering therapy (folate and Vit B) and ALA supplementation, suggesting that combination therapy may prevent bone loss in PD patients [277]. A combination therapy of ALA and transdermal testosterone provided amelioration of on erectile dysfunction and quality of life in patients with Type 2 DM [59]. Treatment with ALA and γ-linolenic acid was proposed for controlling symptoms and improving the evolution of carpal tunnel syndrome [67]. Antioxidant supplementation with Vit C, Vit E and ALA was reported as a safe, though ineffective, treatment for dementia in individuals with Down syndrome [86].

Combined supplementations were tested in liver disorders, such as chronic hepatitis virus C infection. Melhem *et al.* [72] reported on a combination of ALA with antioxidative preparations (glycyrrhizin, *Schisandra*, silymarin, Vit C, l-glutathione, and Vit E) in patients with chronic hepatitis C, with favorable response rate.

Twelve clinical trials have reported on combination regimens with CoQ10 and various antioxidants (Table 6). Witte *et al.* [112] tested a combination of CoQ10 and high-dose micronutrients that was found to improve left ventricular function and quality of life in elderly patients with chronic heart failure. Shargorodsky *et al.* [128] tested a combination therapy with CoQ10, Vit C, Vit E and selenium in patients with multiple cardiovascular risk factors, finding an improvement in glucose and lipid metabolism, and decreased blood pressure.

**Table 5:** Clinical studies utilizing mitochondrial nutrients and antioxidants and/or herbal compounds aimed at compensating OS/MDF-related pathogenetic mechanisms.

Diseases/Conditions	α-Lipoic Acid + Other Agent(s) (Daily Dose)	References
Type 1 and Type 2 diabetes	ALA 100 mg, CoQ10 60 mg, Vit E 200 mg	[41]
	ALA 600 mg, Vit E 800 mg	[44]
	ALA 800 mg, pyridoxine 80 mg	[56]
	ALA 600 mg, transdermal testosterone 50 mg	[59]
	ALA 2 × 600 mg, allopurinol 300 mg, nicotinamide 2 × 750 mg	[60]
Heart surgery	ALA 100 mg, CoQ10 100 mg, Mg orotate 400 mg, ω-3 PUFA 300 mg, Se 200 µg	[81]
Metabolic syndrome	ALA 600 mg, Vit E 100 IU	[77]
Cancer-related anorexia/cachexia	ALA 300 mg, polyphenols 400 mg, carbocysteine 2.7 g, Vit E 400 mg, Vit A 30,000 IU, Vit C 500 mg, (*n*-3)-PUFA 2 cans	[94]
Alzheimer disease	ALA 900 mg, CoQ10 400 mg, Vit C 500 mg, Vit E 800 IU	[65]
Parkinson’s disease	ALA 1200 mg, folate 5 mg, Vit B12 1500 µg	[277]
Carpal tunnel syndrome	ALA 600 mg, γ-linolenic acid 360 mg, Vit B6 150 mg, Vit B1 100 mg, Vit B12 500 µg	[67]
Down syndrome	ALA 600 mg, Vit E 900 IU, Vit C 200 mg	[86]
HCV infection	ALA 300 mg, glycyrrhizin 1 g, *Schisandra* 1.5 g, silymarin 750 mg, Vit C 6 g, l-glutathione 300 mg, Vit E 800 IU	[72]
Psoriasis	ALA 600 mg, CoQ10 50 mg, resveratrol 20 mg, Vit E 36 mg, Krill oil 300 mg, Vitis vinifera seed oil 30 mg, Se 27 mg	[76]
Osteoporosis	ALA 2 × 300 mg, Vit C 30 mg, Vit E 5 mg, Se 2.75 mg	[99]
Vitiligo	ALA 100 mg, Vit C 100 mg, Vit E 40 mg, PUFA 12%	[98]

**Table 6:** Clinical studies utilizing mitochondrial nutrients and antioxidants and/or herbal compounds aimed at compensating OS/MDF-related pathogenetic mechanisms.

Diseases/Conditions	Coenzyme Q10 + Other Agent(s) (Daily Dose)	References
Heart surgery	CoQ10 100 mg, ALA 100 mg, Mg orotate 400 mg, ω-3 PUFA 300 mg, Se 200 µg	[81]
Chronic heart failure	CoQ10 150 mg, Ca 250 mg, Mg 150 mg, Zn 15 mg, Cu 1.2 mg, Se 50 µg, Vit A 800 µg, thiamine 200 mg, riboflavin 2 mg, Vit B_6_ 200 mg, folate 5 mg, Vit B_12_ 200 µg, Vit C 500 mg, Vit E 400 mg, Vit D 10 µg	[112,124]
Cardiovascular diseases	CoQ10 120 mg, Vit C 1 g, Vit E 400 IU, Se 200 µg	[128]
Cardiovascular mortality	CoQ10 200 mg, organic Se 200 μg	[136]
Breast cancer	CoQ10 100 mg, riboflavin 10 mg, niacin 50 mg	[175]
	CoQ10 90 mg, Vit C 2850 mg, Vit E 2500 IU, β-carotene 32.5 IU, Se 387 mg, γ-linolenic acid 1.2 g, *n*-3 fatty acids 3.5 g	[176]
	CoQ10 300 mg, Vit E 300 IU	[177]
Prostate cancer	CoQ10 200 mg, Vit C 750 mg, Vit E 350 mg, Se 200 µg	[179]
Friedreich ataxia	CoQ10 400 mg, Vit E 2100 IU	[140]
	CoQ10 600 mg, Vit E 2100 IU	[141]
Fibromyalgia	CoQ10 200 mg, Gingko biloba extract 200 mg	[160]

A clinical trial utilizing CoQ10 and a mixture of antioxidants was reported by Hertz and Lister [175] to improve survival of patients with end-stage cancer. An early report by Lockwood *et al.* [176] found that supplementation with CoQ10 and antioxidants, γ-linolenic and *n*-3 fatty acids significantly improved clinical conditions in breast cancer patients. A trial by Premkumar *et al.* [177], also conducted on breast cancer patients, reported that a supplementation with CoQ10, riboflavin and niacin decreased the levels of pro-angiogenic factors and increased the levels of anti-angiogenic factors, and reduced tumor burden. On the other hand, a combination therapy with CoQ10 and Vit E did not result in improvement of fatigue symptoms in breast cancer patients, according to Lesser *et al.* [178]. Negative results were obtained by Hoenjet *et al.* [179] by testing a combined supplement containing CoQ10, Vit E, Vit C and selenium in patients with hormonally untreated carcinoma of the prostate, which did not affect serum level of PSA or hormone levels.

Two studies by Hart and co-workers [140,141] reported on a combined treatment with CoQ10 and Vit E in patients with Friedreich ataxia finding sustained improvement in mitochondrial energy production and a significant improvement in cardiac function. A combination therapy with CoQ10 and *Ginkgo biloba* extract significantly improved quality-of-life in patients with clinically diagnosed fibromyalgia [160].

Taken together, the literature on clinical trials testing MN and “classical” antioxidants may provide crucial data on the therapeutic efficacy of compounds aimed at improving mitochondrial health and several OS/MDF-related endpoints, suggesting the design of improved treatments of OS/MDF-related diseases.

## 7. State-of-Art: Critical Remarks

The present survey of published clinical trials testing MN may reflect the current state-of-art across broad-ranging disorders that are usually focused in separate medical disciplines. On the other hand, the clinical trials testing MN published to date have been conducted on some selected diseases, far below the potential scope of investigation, by considering the extensive range of OS/MDF-related disorders [9]. Trying to interpret this delay, one might find one or more of the following explanations: (a) a disease is commonly recognized to rely on other etiologic grounds than OS/MDF: this is the case, e.g., of Fanconi anemia and of other genetic diseases [8,278]; (b) very rare diseases discourage patient recruitment in view of clinical trials as, e.g., progerias [8,279]; (c) the specialist community may be committed in established therapeutic strategies, thus disregarding potential adjuvant interventions.

Other two remarks may be addressed to the state-of-art in the literature of clinical trials using MN. First, the reviewed studies were mostly oriented to clinical endpoints of the investigated disorders, by underscoring any relevant mechanistic aspects enabling to elucidate trial’s results. Second, and most relevant, the vast majority of the reviewed trials tested one MN only, by disregarding: (a) the complementary roles of these cofactors in mitochondrial functions; (b) their different physico-chemical behaviors in water- *vs.* lipid-phase; and (c) the scarce, if any, knowledge as to specific MDF in a given disorder and the appropriate requirements of ALA, or CoQ10, or CARN (or its derivatives) supplementation. Thus, the as yet prevailing design of trials using only one MN may fail to achieve the desired outcomes of OS/MDF compensation and hence of improving clinical endpoints. This limitation, along with some possible shortage in dosages and/or in trial duration might have contributed to some unsuccessful trial outcomes. One may note that supplement combinations of more than one MN, or the use of ALA or CoQ10 with classical antioxidants may have contributed to the overall success of these trials (see Table 4, Table 5 and Table 6). Thus, one may anticipate the optimization of future clinical trials in OS/MDF-related disorders by means of combined MN treatments, possibly associated with “classical” antioxidants.

The choice of MN formulations may have critical implications on the expected success of MN-based clinical trials. An outstanding aspect in the design of a MN-based clinical trial is represented by the type of formulation chosen. The oxidative state of molecules, chirality, and the vehiculants used in the formulation may have dramatic implications in terms of bioavailability and interaction with the recipient biological system. In the case of CoQ10, bioavailability is known to be influenced by modality of administration, with higher plasma levels reached by administration of the same amount divided in multiple doses, and by vehiculant type with soft gels containing triglyceride dispersing medium showing a better bioavailability in comparison with the crystalline form [280]. Another issue, also linked with bioavailability, is the oxidative state of the active compound in the formulation. Most of the studies cited used ubiquinone, however recently also the reduced form of CoQ10 (ubiquinol) has been used in several clinical trials [113,114,198]. Direct use of ubiquinol displays the advantages of improved absorption and consequently enhanced bioavailability, and direct exposure of the reduced (and active) antioxidant form to digestive epithelial tissue; finally, ubiquinol may influence the ubiquinone/total CoQ10 ratio in conditions where reductive systems are sub-optimal as it has been shown in the aging process [281].

α-Lipoic acid is characterized by an enhanced bioavailability compared to CoQ10, with absorption rates around 30%–40% of an oral dose of ALA [282]. Moreover, while ALA being synthethized by biological systems is exclusively in the *R*-lipoic acid chiral form, formulations are often (*R*/*S*) or (+/−) ALA, composed by a 50/50 mixture of *R*- and *S*-ALA. It has been shown that bioavailability of the two isomers is different, *R*-lipoic acid showing peak plasma concentrations in pharmacokinetic studies 40%–50% higher than *S*-LA, suggesting that *R*-LA is better absorbed than *S*-LA [282].

Regarding CARN and its derivatives, especially ALC, the state-of-art provides extensive information as to the efficacy of CARN, ALC and PLC. The advantages of using ALC *vs.* CARN have been provided [200,201,202,203], along with successful use of ALC [68,78,222,223,226,231,246,247,248,252,253,254,261,266,267,268] or PLC [210,225,235,239,253,272] in a number of clinical trials. Acetyl-l-carnitine probably acts as an acetylating agent and its involvement in epigenetic modifications of proteins might explain its success in aging and in OS/MDF-related disorders [283,284,285]. Future research on CARN/ALC/PLC supplementation should address the correlation of supplement dosage, changes and maintenance of tissue concentrations, and metabolic and functional changes and outcomes [286,287]. An analogous research strategy was highlighted by Camp *et al.* [288] on nutritional interventions for inborn errors of metabolism, suggesting combined strategies in improving the clinical management of an extensive number of disorders.

## 8. Prospects of Clinical Trials in OS/MDF-Related Disorders

**Figure 1:**
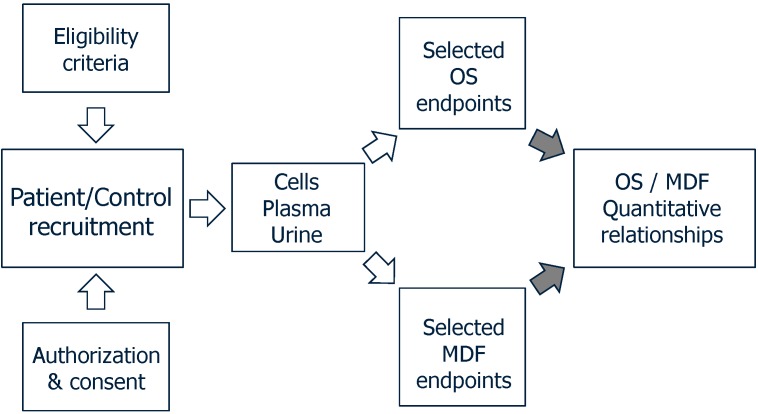
Preliminary database to be achieved in design of mitochondrial nutrients (MN)-based clinical trials.

The scheme depicted in Figure 1 points to the prerequisite of identifying the OS and MDF endpoints that are altered in a given disorder, often incompletely elucidated. A choice of OS endpoints should include some established parameters including oxidative damage to DNA, lipids, carbohydrates and proteins, and changes in the glutathione system. A few selected MDF endpoints should be addressed to verify the deficiency, if any, of ALA (to date almost unexplored), CoQ10, and CARN. Other relevant endpoints in assessing an *in vivo* MDF should include mitochondrial DNA content, ATP production, OXPHOS activities, lactate/pyruvate ratio, and expression of mitochondrial antioxidant activities (MnSOD and peroxiredoxin 3). This set of measurements should clarify the quantitative aspects of the disorder to be focused in a clinical trial design. Once the characterization of OS/MDF endpoints is accomplished, the target outcomes of the clinical trials may be defined.

**Figure 2:**
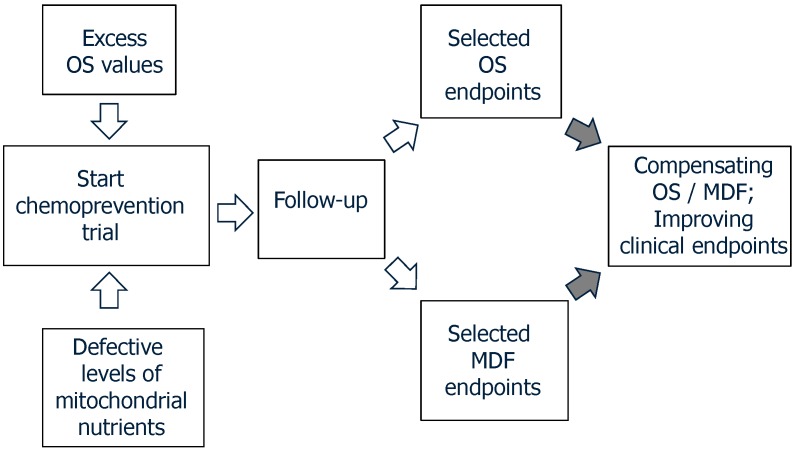
Follow-up procedures combining evaluation of MDF and OS endpoints, along with disease specific clinical endpoints.

As shown in Figure 2, the OS/MDF endpoints for a given disease, found as abnormal in the preliminary step, shall be the grounds for choosing the best appropriate supplements to be administered to patients. These supplements may include one or—more reasonably—more MN, as discussed above. The follow-up period will be of paramount relevance; indeed, short admnistration regimens may offer hints for efficacy and safety, yet they may conceal long-run effects. Moreover, in diseases with multi-year-long progression, expecting the desired outcomes may be a quite elusive task. Thus, follow-up ought to be designed for a reasonably long duration with, e.g., six-month intervals in results analysis, allowing us to monitor the outcomes of multi-year-long administration. The ultimate goal will be achieved by compensating OS/MDF endpoints, along with the amelioration of disease progression.

Materializing the above prospect may have dramatic consequences in the success of to-be clinical trials for OS/MDF-related disorders, trespassing from “hopes”, or from single-parameter information, toward a rational approach that shall foresee properly targeted interventions, then with rational expectations, rather than attempts.
